# Effectiveness of digital and analog learning methods for learning anatomical structures in physiotherapy education

**DOI:** 10.1186/s12909-024-05484-1

**Published:** 2024-05-06

**Authors:** Larissa Pagels, Robert-Christopher Eschke, Kerstin Luedtke

**Affiliations:** Pain and Exercise Research Luebeck, Institution of Health Sciences, University of Luebeck, Luebeck, Deutschland

**Keywords:** Anatomy, Learning, Digital, Hybrid, Physiotherapy

## Abstract

**Background:**

According to the German Physiotherapy Education and Qualification Regulations, teaching of anatomical structures is one of the fundamental subjects of physiotherapy education. Besides exhibits and models, anatomy atlases are usually used as teaching and learning tools. These are available in both analog form such as printed books or in digital form as a mobile application. Furthermore, the use of digital teaching and learning tools is steadily increasing within the education of health professionals.

**Aim:**

To assess the efficacy of a digital educational tool in contrast to an analog anatomical atlas in acquiring knowledge about anatomical structures.

**Material and method:**

The data collection took place in the context of an anatomy tutorial for students of the bachelor’s degree program in physiotherapy. In a cross-over design, the students completed two learning assignments, each, with different learning materials provided, either with an anatomy app on a tablet or with an anatomy atlas as a book. The tests to assess the newly acquired knowledge immediately after the task, consisted of questions about the anatomical structures of the knee as well as the shoulder. In addition, the students’ satisfaction with the learning materials provided was surveyed using a questionnaire. The survey assessed their satisfaction, their assessment of learning success, and their affinity to digital learning materials. This was done using a 5-point Likert scale and a free-text field. The data was analyzed descriptively, and group differences were calculated using a t-tests.

**Results:**

Thirty students participated. The group comparison showed a significantly better outcome for the group that prepared with the analog anatomy atlas for the questions on the knee than the comparison group that used the anatomy app (t(28) = 2.6; *p* = 0.007). For the questions concerning the shoulder, there was no significant difference between the digital and analog groups (t(28) = 1.14; *p* = 0.26). The questionnaire revealed that satisfaction with the analog anatomy atlas was significantly higher than with the anatomy app. A total of 93.34% rated their experience with the analog learning tool at least “somewhat satisfied”. In contrast, 72.67% of students partially or fully agreed that they “enjoyed learning with digital learning tools”.

**Discussion:**

Learning anatomical structures with the Human Anatomy Atlas 2023 + app did not show a clear advantage when compared to an anatomy book in these two cohorts of physiotherapy students. The results of the questionnaire also showed greater satisfaction with the analog anatomy atlas than with the anatomy app, whereas most students stated that they frequently use digital learning tools, including some for anatomical structures. Satisfaction with the learning tool seems to play a central role in their effectiveness. In addition, sufficient time must be provided for users to familiarize themselves with the user interface of digital applications to use them effectively.

**Registration:**

Diese klinische Studie wurde nicht in einem Studienregister registriert.

## Introduction

The desire for digital teaching is growing in all areas of university teaching. Due to the Sars-CoV-2 pandemic, teaching has experienced a major boost in digitalization since March 2020 [1]. A substantial number of digital teaching and learning tools are already available for this purpose. Especially in the of basic education in health sciences, the use of various teaching and learning tools can promote student motivation [2].

As part of the “HySkiLabs project - Teaching and learning health in hybrid skills labs”, education in the health sciences at the University of Luebeck is being enriched with digital teaching and learning tools. The project not only aims to transfer classroom teaching approaches to digital and hybrid teaching-learning environments, but also to systematically investigate their effectiveness. Previous surveys have shown a positive attitude of students towards digital teaching [3].

Over the last few years, the availability of digital learning tools has also increased considerably. There are various softwares and apps that students can use to organize their time in self-study or that are used as complementary teaching methods in anatomy lectures. An early study by Keedy et al. (2011) compared a 3D digital application to a 2D form for learning the anatomy of the liver and gall bladder by medical students [1]. No significant difference between the two visually different teaching methods was found for the knowledge of anatomical structures at the end of their study. Nevertheless, students’ satisfaction with the 3D digital application was very high. Two years later, Noguera et al. (2013) analyzed the effect of a digital 3D anatomy app in comparison to traditional teaching (lectures) in a physiotherapy degree program [2]. They found significantly better results in musculoskeletal anatomical knowledge among those students who used the digital anatomy app. More recently, Browne et al. (2019) analyzed the effect of online quizzes to learn anatomical structures complementing traditional learning in laboratory sessions (with wet and dry specimen, plastic models, histological slides etc.) and lectures [3]. Questions using images of anatomical structures and multiple-choice questions were provided in the online quizzes that were subsequently completed by students during self-study periods. The experiences of the students were evaluated and indicated a high level of engagement and satisfaction with the supplementary online material. Another study from 2014 used online discussion forums as an addition to their traditional learning (laboratory sessions and lectures) as an option for the students to interact and help each other in the learning process [4]. This digital learning method showed good effects on the students grades at the end of the module. In 2023, a study used Kahoot! quizzes to promote the learning of anatomical structures with a game-based learning method [5]. The quizzes contained questions about anatomical structures with four response options and were presented at the end of each lecture. An open-book technique was used, giving the students only 20s to answer the questions. A significant increase of short-term knowledge retention and an increase in the frequencies of correctly answered responses was found, compared to the traditional teaching method (lectures without Kahoot! quizzes). Additionally, all students perceived that the use of the interactive quiz improved their anatomy short-term knowledge retention.

Innovative computer-based learning tools can improve the learning of the complex spatial relationships of the musculoskeletal system and facilitate the transfer of anatomical knowledge to patients [5]. Inaccurate identification of anatomical structures is a common source of error in the assessment and treatment of musculoskeletal conditions, therefore, accurate learning of these, is essential for clinical practice [5]. From an educational perspective, interactive learning with 3D visualizations also offers several potential advantages over traditional methods of teaching anatomy: (1) a directly recognizable visualization of anatomical structures, (2) a reduction in cognitive load as students do not need to build their own mental visualization of the model, (3) many different anatomical perspectives and the ability to move the model interactively, and (4) the ability to incorporate 3D models obtained from live human imaging datasets − 2D drawings of anatomical structures are potentially inaccurate [5, 6].

In this study, a digital 3D anatomy atlas was used to promote the short-term learning retention of physiotherapy students. To create a comparison between an analog and digital learning tool in this study, the app Human Anatomy Atlas 2023 + by Visible Body® (further referred to as “digital anatomy app”) was chosen.

There are currently no studies to indicate how effective this digital teaching and learning tool (digital anatomy app) is compared to traditional methods (analog anatomy atlas), hence, this study investigated the effect of using the *Human Anatomy Atlas 2023 +* on physiotherapy students’ learning of anatomical structures compared to learning with the *Prometheus Atlas of Anatomy (further referred to as “analog anatomy atlas”)*.

## Methods

The study was designed as an empirical cross-sectional study. The data collection took place in the context of a tutorial in which students were able to intensively study anatomical structures of the musculoskeletal system and peripheral nervous system. For this purpose, they were using work assignments, as well as teaching and learning tools provided by the supervisor.

**Fig. 1:**
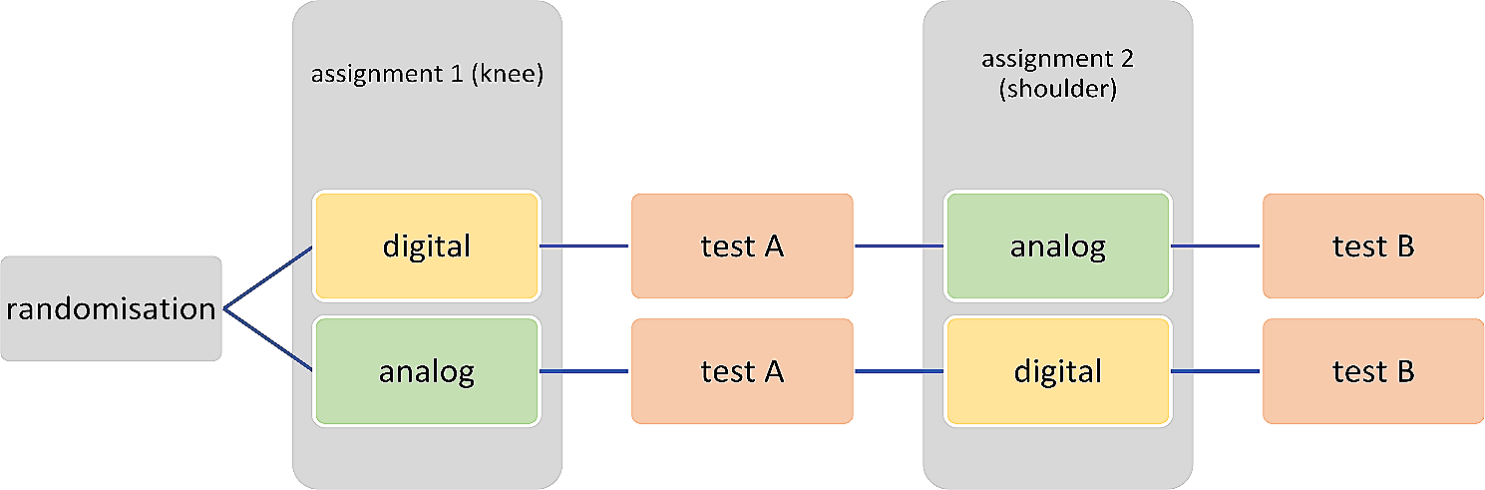
Study design

The students were randomized into two groups (“digital/analog” or “analog/digital” depending on the order of learning tools that were provided) and allocated by one of the supervisors of the tutorial in two different rooms before the beginning of the study. The groups attended the tutorials in these two different rooms and were both given the same tasks but with different teaching and learning materials (Fig. 1).

### Participants

All students of the bachelor’s degree program in physiotherapy at the University of Luebeck were invited to participate in the study. At the University of Luebeck the anatomy module is taught as face-to-face lectures, practice sessions in the dissection room and 50% self-study time. In the latter, the students deepen their knowledge independently - typically this is done with the help of anatomy atlases. These can be analog (2D) and digital (2D or 3D). As a rule, the collective work “Prometheus- Atlas of Anatomy” [4] serves as an analog anatomy atlas. Previous knowledge of anatomical structures was mandatory for the participation in this study, but it had to be assumed that the knowledge was rather heterogenous due to different levels of studying of the participants. The students were informed about the data collection at the beginning of the tutorial and written consent was obtained. Participation was voluntary and had no influence on the tutorial procedure, further study program or examination results.

### Application used in this study

There are several applications to learn anatomical structures with different learning modes. Some show theoretical descriptions, as well as drawings (2D) of anatomical structures, and additional skill related content as placing of ultrasound probes or manual palpation techniques (Ecofisio app; [6, 7]). Other applications use vision-based augmented reality to display anatomical structures on human models [8] or in the room, with the option to move around the augmented reality simulated anatomical structure [8, 9]. In addition to augmented reality 3D visualizations of anatomical structures, there are also applications that use three-dimensional images to display their content interactively [2, 9].

The digital anatomy app used for the purpose of this study (Human Anatomy Atlas 2023+), provides various options to learn anatomical structures, and physiological processes using 3D models (by option as augmented reality simulation). The learning content is presented as a 3D model, which is interactive and can be used individually by the students. Thereby, various information on the anatomical structures and common pathologies can be accessed and learned. Additionally, short videoclips of functional anatomy (e.g. showing the muscles that are required to bend the knee while an animated skeleton is bending the knee), or rather complex functions as swallowing food, are part of the content of the app. The app does not provide options for self-testing of knowledge. This app was chosen after screening different options as it is already known by some of the students and the teaching staff and it offers interactive 3D models that have been proven to facilitate knowledge gain and satisfaction of the students when learning anatomical structures [2, 8]. Next to being the most practicable option (as it needs time to familiarize with the interface of new applications) this app provides all functions needed to operate with the work assignments in this study. The costs of the app were covered by the “Stiftung Innovation in der Hochschullehre” as part of the HySkiLabs project.

### Material and procedure

Work assignments were prepared by the supervisor of the tutorial. These contained questions about structures of the knee (ligaments, bone and joint structures) including surrounding muscles and their innervation (assignment 1). Assignment 2 focused on the shoulder joint. The supervisor was a physiotherapist with experience in teaching and good knowledge of anatomical structures. Both work assignments were double-checked by faculty members of the physiotherapy degree program of the University of Luebeck for comprehensibility.

The knowledge of the students after each learning session, was assessed via written tests that contained open ended questions about the previously repeated learning content (e.g. “name all the ligaments of the knee joint and their special features.“). The number of points to be achieved were displayed next to each question, so that the students knew about the expected scope of the answers.

In the group “analog/digital”, each participant received an analog anatomy atlas (Prometheus), while in the group “digital/analog”, each participant received the digital anatomy app on a tablet device (Human Anatomy Atlas 2023+). All students were given an initial 45-minute work assignment, which was identical in both groups and related to structures of the knee joint. A supervisor was available in each room to answer questions.

to familiarize themselves with their learning tool. Merely a verbal suggestion was given to the users of the anatomy app to use the search function of the app. After the first assignment, the participants completed the first test (maximum score 41 points) on the teaching content. During the test, no books or apps were allowed. Afterwards, the teaching and learning materials were exchanged in the rooms and the participants thus received the respective teaching and learning tool. With the new teaching and learning tool, the participants worked on another 45-minute assignment (on structures of the shoulder joint) and completed the subsequent test (maximum score 47 points).

Subsequently, the students filled out a questionnaire in which their name, age, gender and satisfaction with the teaching/learning tool offered (0 = not at all satisfied − 5 = very satisfied) were asked. The teaching/learning tool used privately by the students (free response option) and the desire for similar teaching units as exam preparation (0 = not at all − 5 = absolutely) on a 5-point Likert scale were also part of the questionnaire.

In addition, the following sub-questions were formulated for secondary analyses and assessed as a survey by students after the completion of the tasks:

- How satisfied are students with the analog or digital teaching and learning tools measured on a 5-point Likert scale?
- How do students rate their learning success in relation to the teaching and learning tools available on a 5-point Likert scale?
- Are the teaching and learning tools offered known and have they already been used by the students (open ended question)?

### Data analysis

The collected data were tabulated and analyzed using Stata (Student Version BE 17, Mac).

The null hypothesis for the analysis was:

H0 = there is no difference between the group using an analog anatomy atlas and the group using a digital anatomy app.

H1 = the respective group that learns with the digital anatomy app shows better results in the tests.

Socio-demographic data, answers from the questionnaire and the evaluation of the work assignments were analyzed descriptively with regard to frequencies (mode, median, mean) as well as dispersion measures ((interquartile) range, standard deviation) and shape measures (kurtosis, skewness) for the groups “analog/digital” and “digital/analog”.

Normal distribution of the data was tested using Shapiro-Wilk tests and group differences were calculated using t-tests.

The assessment of the group differences took place on the basis of the calculated Cohen’s d. Thus, the effect size of the use of digital vs. analog teaching and learning aids (here: anatomy atlases) was determined.

## Results

Thirty students from the semesters 2–8 of the physiotherapy degree program of the University of Luebeck participated in the study. The demographic analysis revealed an asymmetric data set for the variable semester in the analog/digital group and the variable age in the digital/analog group. The detailed results can be found in Table 1. The majority of participants identified as female (*n* = 25; 83.3%). The groups analog/digital and digital/analog differed significantly in the distribution of male and female participants (t(28)=-2.43; *p* = 0.01).

**Table 1:** Demographic characteristics with significance of group differences

	group		significance
	**analog/digital (** ***n*** ** = 14)**	**digital/analog (** ***n*** ** = 16)**	
gender (female; n (%))	14 (100)	11 (68,75)	0.01
age (M, SD)	21 (1,71)	22,13 (4,72)	0.41
semester (M, SD)	3,57 (2,1)	3,5 (1,71)	0.92

**Fig. 2:**
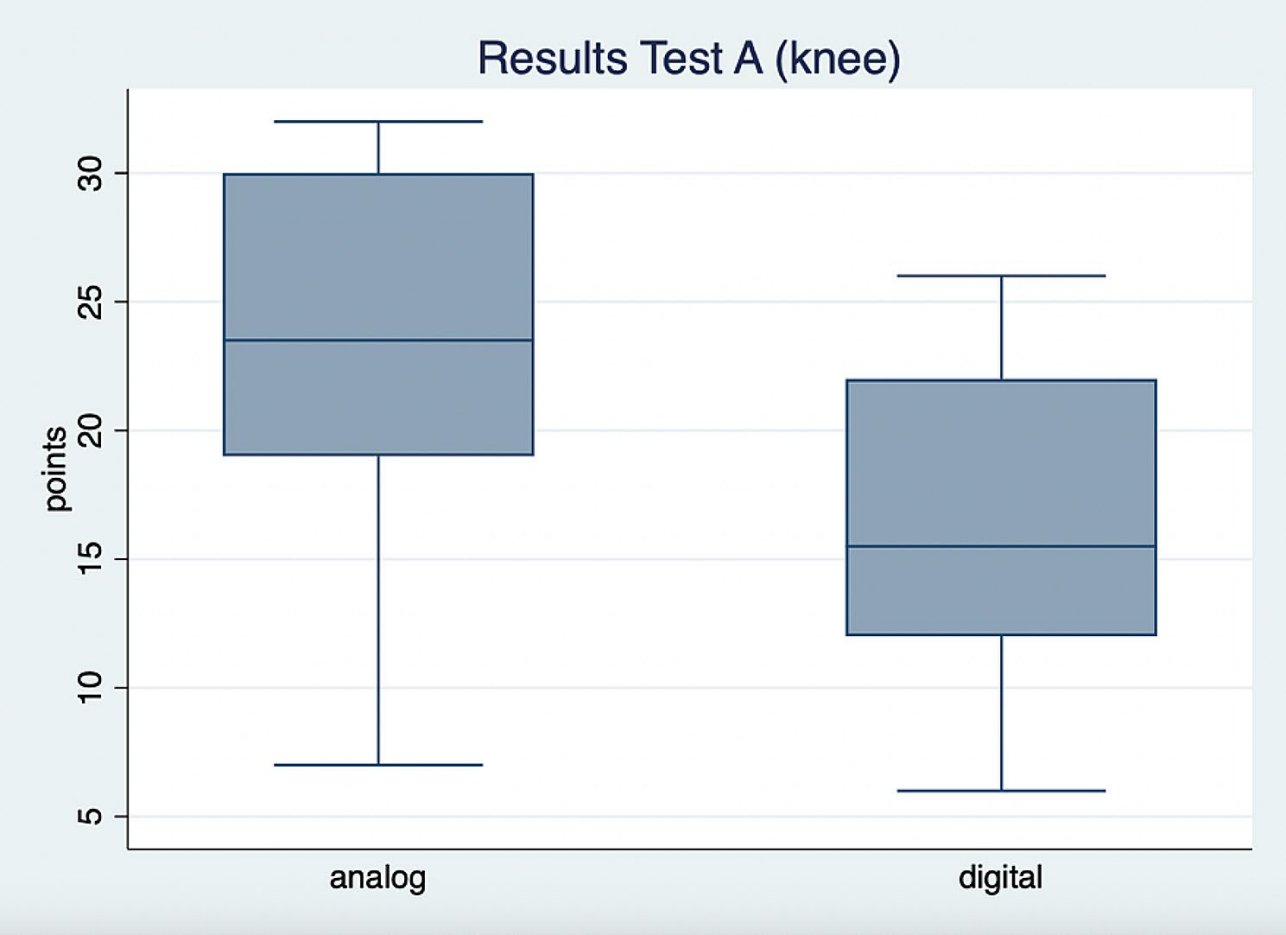
Boxplot of the results of test A

**Fig. 3:**
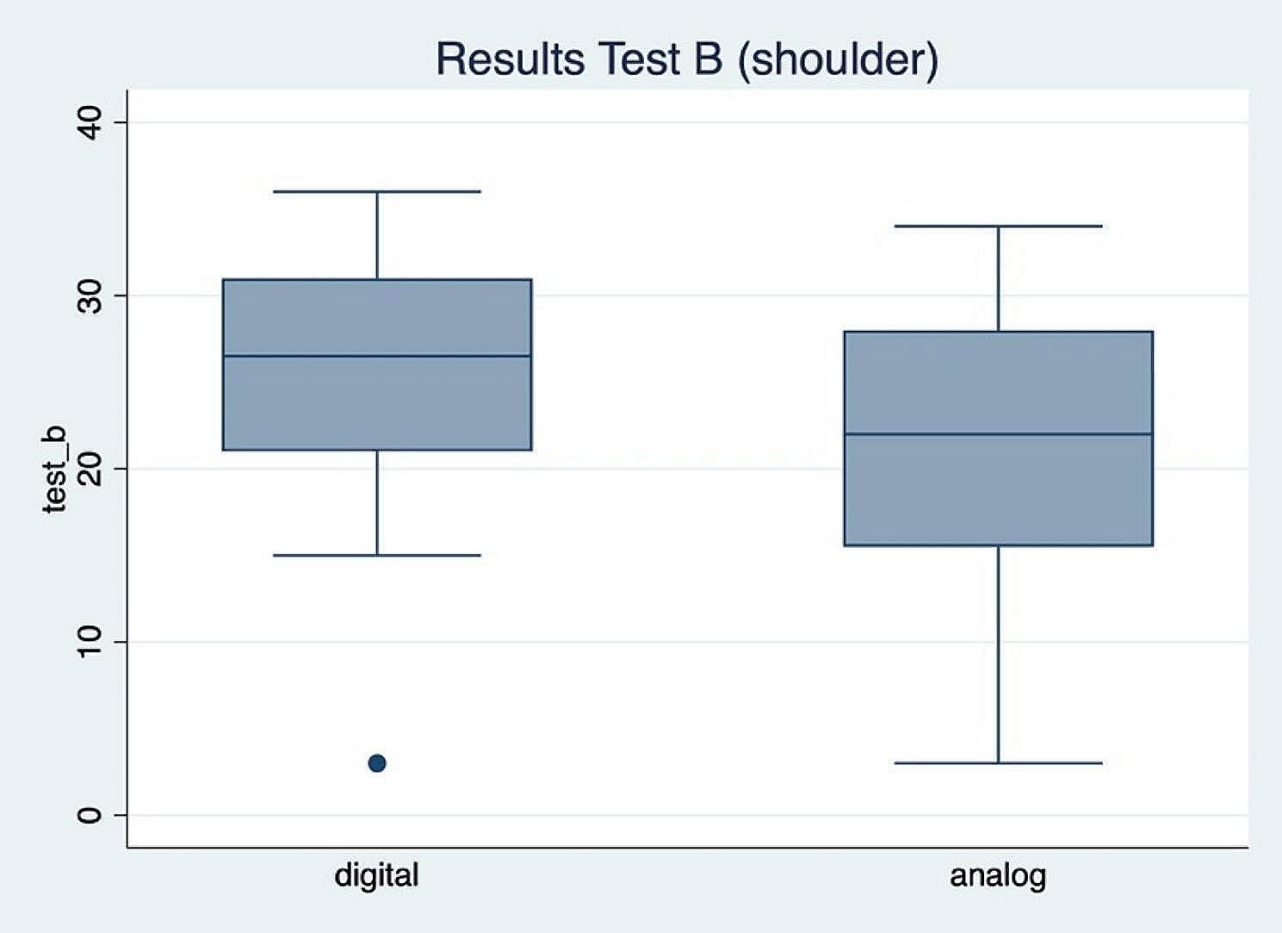
Boxplot of the results of test A

The results of test A (knee) unveiled a significant group difference (t(28) = 2.6; *p* = 0.01) and a Cohen’s d of 0.95 (Fig. 2). with a higher score for the group that completed the task using an analog anatomy atlas. No significant group difference was found for test B (shoulder) (t(28) = 1.14; *p* = 0.26). In that analysis the effect size was a Cohen’s d of 0.42 (Fig. 3; Table 2).

**Table 2:** Results of the written exams with significance of group differences

		group		significance
		**analog/digital (** ***n*** ** = 14)**	**digital/analog (** ***n*** ** = 16)**	
test A (knee) (M, SD)		23,36 (7,56)	16,63 (6,61)	0.01
test B (shoulder) (M, SD)		24,79 (8,89)	21,13 (8,7)	0.26

The evaluation of the questionnaire (Table 3) showed that satisfaction with the analog anatomy atlas is significantly higher than with the anatomy app. In the question about the analog learning tool 93.34% selected “somewhat satisfied” to “very satisfied”. On the other hand, 43.33% of the participants were “somewhat dissatisfied” with the digital anatomy atlas offered. In contrast, 72.67% of the students partially or fully agreed that they generally “enjoy learning with digital learning tools”.

**Table 3:** Results of the questionnaire (percentage of participants who answered in each category of the 5-point-Likert scale)

	not at all satisfied	somewhat dissatisfied	neutral	somewhat satisfied	very satisfied
How satisfied were you with the analog teaching and learning tools offered to you?	-	3,33	3,33	56,67	36,67
How satisfied were you with the digital teaching and learning tools provided to you?	-	43,33	26,67	20	10
	**disagree**	**rather disagree**	**neutral**	**rather agree**	**agree**
I enjoy learning with digital teaching and learning tools.	3,33	3,33	16,67	50	26,67
I see a greater learning effect when learning with digital teaching and learning tools than with analog ones.	6,67	26,67	33,33	23,33	10

Twenty of the students stated that they learn privately with the analog anatomy atlas used in the study (Table 4). In addition, the students mainly use the notes from the anatomy lectures (*n* = 12), and the lecture material (*n* = 10). Apps and software for learning anatomical structures, on the other hand, were mentioned less frequently. Only 10 of the students stated that they used additional software (Visible Body, Anvil, etc.) for independent learning of anatomical structures. It was frequently mentioned that the Prometheus atlas was used digitally and recordings from the lectures and other teaching videos were used.

**Table 4:** Usage of different educational tools for the anatomy course

	*n*	Teaching/learning tool
What teaching and learning materials do you use privately to study for the anatomy module?	20	Prometheus (analog or as PDF)
	12	Own notes from lectures/notes from previous semesters
	10	Lecture material/slides from the university/scripts
	3	Other learning books
	3	Study groups
	3	Index cards
	9	Amboss
	8	Atlas-App
	6	Google/Internet
	4	DocCheck
	3	Learning videos
	3	Anki
	1	Anatomie-Quiz-App
	1	Kenhub

## Discussion

This study analyzed the effect of learning anatomical structures with a digital anatomy app in comparison to the use of an analog anatomy atlas in the context of a physiotherapy students’ tutorial. The test results showed that the group which prepared with the analog anatomy atlas for the first test (A; knee) performed significantly better than the digital group. This could not be confirmed with the second test (B; shoulder). Hence, the results of this study about the effect of the digital anatomy app on knowledge gain is ambivalent.

Two-thirds of the participants (*n* = 20) reported that they used the analog Prometheus Anatomy Atlas for studying at home and expressed satisfaction with it as a learning tool during the tutorial. Interestingly, the questionnaire also revealed that the students enjoy working with digital learning tools, but not with the one they used during the study. This might explain the difference in the first test results because it insinuates that the students had a better learning experience with the familiar learning tool. This can be supported with the results of a study from 2016, that was able to show that familiarities improve the acquisition of new knowledge. This can also be supported by the fact that the app can be used more effectively if the user interface is known beforehand and operation are clear because less working memory is devoted to understand the interface [10, 11].

Research has shown that students’ dissatisfaction with a learning tool plays a role in its effectiveness and learning success [2]. As the participants in this study were not satisfied with the digital anatomy app provided (Table 2), this presents a valid explanation for the poor results of the digital learning outcomes. Moreover, many students of this sample still prefer to use analog learning materials (e.g. index cards, lecture notes, Prometheus atlas) or combine both by supplementing their analog learning materials with information from the internet (e.g. DocCheck, learning videos), further explaining the results.

The main functions of the digital anatomy app used in this study are of interactive nature and it is assumed, that active learning took place when students used the app. Interactive learning has been shown to lead to greater learning progress [12]. There is evidence for a significant better knowledge gain and student satisfaction when learning anatomical structures with mobile applications compared to traditional learning (2D images, textbook learning) [2, 6–8]. The fact that the test results did not show a superiority of the digital learning tool compared to the analog anatomy atlas could be, amongst other things, that the students had too little time to learn the body regions tested digitally. But according to Noguera et al. (2013), it is necessary that students have enough time to internalize anatomical structures learned in 2 dimensions and to convert them into a 3D understanding as well [2].

Previous studies have found divergent results when comparing digital vs. analog learning tools for learning anatomical structures. Keedy et al. already showed in 2011 that there was no significant difference in learning anatomical structures (liver and gall bladder) with a 3D digital application or a 2D application [1]. In contrast to the present study, the students’ satisfaction with the 3D digital application was very high [1]. One reason might be that in 2011 there were fewer alternative digital learning tools available and the comparison between several digital learning tools was therefore low.

Contradicting tothe present results Noguera et al. (2013) found a significantly better result in musculoskeletal anatomical knowledge among physiotherapy students who used a digital (3D) anatomy app than among students who received traditional teaching [2]. This difference may be attributed to their utilization of a different, more rudimentary application, characterized by a reduced set of functions compared to the alternative. Presumably, this helped students to familiarize themselves with the digital application more quickly leading to better learning effects. Furthermore, anatomical knowledge was tested witha multiple-choice questionnaire, which means that the mere probability of correct answers is higher than in this study.

### Limitations

In this current study, only the students’ ability to acquire knowledge in a short time and to recall it immediately, is tested. No conclusion can be drawn about how well the students can recall the knowledge acquired after a longer period of time. Likewise, it is not possible to say how good the students’ knowledge was in advance of the tutorial, so that the learning gain through the work assignments cannot be precisely mapped. Since it was announced in advance of the tutorial that the test results would have no effect on the further course of studies, students might not have taken the test seriously. However, this effect would have been comparable in both groups.

The survey used in this study was only checked by faculty members for comprehensibility, relevance, expected acceptance of the students as well as feasibility. It has not been pilot-tested in the target population (physiotherapy students), therefore no conclusion can be drawn to its content validity.

## Conclusions

This study highlights that the analog and familiar learning tools are superior if the user-friendliness and simplicity of the digital tool are not on a comparable level. Regarding the “HySkiLabs” framework project, it can be deduced from the results that the students enjoy working with digital learning tools, but a higher effectiveness of these tools could not be shown.

Further research should investigate, whether additional teaching and learning methods like discussion forums, or interactive quizzing situations might be more beneficial for knowledge retention of anatomical structures and enjoyment of learning than the mere tool itself [3–5].

Through digitalization, technical solutions are increasingly emerging with the potential to positively effect students’ motivation to learn and provide an effective learning environment [13, 14].

## Data Availability

The datasets used and/or analyzed during the current study are available from the corresponding author on reasonable request.
